# Chemical Profiling and Biological Activities of *Ruta montana* Volatile Oil: *In Vitro* and *In Silico* ADME/Tox Assessment

**DOI:** 10.1155/sci5/6077249

**Published:** 2025-10-14

**Authors:** Nesrine Benkhaira, Naoufal El Hachlafi, Amine Elbouzidi, Mohamed Addi, Saad Ibnsouda Koraichi, Kawtar Fikri-Benbrahim

**Affiliations:** ^1^Laboratory of Microbial Biotechnology and Bioactive Molecules, Faculty of Sciences and Technologies Faculty, Sidi Mohamed Ben Abdellah University, Fes, Morocco; ^2^Faculty of Medicine and Pharmacy, Ibn Zohr University, Guelmim 81000, Morocco; ^3^Laboratory for the Improvement of Agricultural Production, Biotechnology and Environment (LAPABE), Faculty of Sciences, Mohammed Premier University, Oujda, Morocco

**Keywords:** ADME, antimicrobial, antiradical, bioactive compounds, enzyme inhibitors, molecular docking, *Ruta montana*

## Abstract

*Ruta montana* L. is a perennial medicinal herb traditionally used in Moroccan healing practices for infectious diseases, hyperglycemia, spasms, and fever. This work intends to evaluate the bioactive constituents of *Ruta montana* essential oil (RMEO) using the GC–MS technique and demonstrate its antimicrobial, antioxidant, and antidiabetic properties via *in vitro* and molecular docking studies. The primary constituents of RMEO are 2-undecanone (56.41%), limonene (10.28%), and 2-nonanone (8.92%). The α-amylase (IC_50_ = 455 ± 0.11 μg/mL) and α-glucosidase (IC_50_ = 401 ± 0.04 μg/mL) enzymes were significantly inhibited by RMEO. Additionally, ferric reducing power (EC_50_ = 1188.61 ± 1.12 μg/mL) and the β-carotene bleaching assay (IC_50_ = 206.21 ± 1.12 μg/mL) confirmed its strong antioxidant activity. The antimicrobial evaluation showed remarkable activity against *Micrococcus luteus* (IZ = 20.05 ± 0.98 mm), followed by *Staphylococcus aureus* (IZ = 18.11 ± 1.11 mm) and moderate anticandidal activity against *Candida albicans* (IZ = 13.05 ± 0.21 mm). Minimal inhibitory and bactericidal concentrations (MIC and MBC) ranged from 2.25 to 72 mg/mL, confirming both bactericidal and fungicidal effects. Besides, *in vitro* findings were supported by molecular docking and ADME analysis. The present investigation highlights the potent antibacterial, anticandidal, antioxidant, and antidiabetic abilities of RMEO, supporting its potential as a natural and safe therapeutic agent.

## 1. Introduction

Long-term, infectious diseases have posed serious challenges to society systems, economic stability, and global health. The World Health Organization (WHO) reports that these diseases are a major cause of death globally, primarily in low- and middle-income nations [1].

Furthermore, oxidative stress can induce highly oxygen-derived free radicals, including dihydrogen peroxide, oxygen radical, and hydroxyl species, which will damage certain cellular components. According to [2], this damage may lead to significant gene changes, disrupted signaling networks, and reduced cellular functions. This may result in poor health, namely in those who have diabetes. Another of the most important medical and social health issues facing the world today is diabetes mellitus. According to estimates, the prevalence of diabetes mellitus in industrialized countries lies at around 5%–6% and is rising [3]. Hence, there is an urgent need for alternative strategies to face the increasing burden due to such health issues.

Many studies have indicated that medicinal and aromatic plants and trees provide rich sources of bioactive compounds [4, 5]. Essential oils (EOs) obtained from these plants are endowed with a plethora of biological and pharmacological qualities, particularly anti-inflammatory, antiradical, antitumor, and antihyperglycemic activities [6]. Antibacterial, antiviral, and antifungal properties of EOs have also been used to combat microorganisms that cause serious infectious diseases [7, 8]. Thus, perhaps, but to evaluate biological properties of extracts of medicinal plants containing volatile oils rich in bioactive molecules promises to discover novel, safe, and efficient compounds with antimicrobial, antioxidant, and antihyperglycemic effects.

Taking the example of *Ruta montana* (RM), commonly called as mountain rue, is distributed in the Mediterranean country and the Middle East. This prolific herb thrives in rocky and mountainous terrains of this area and is popular for its traditional medicinal uses along with aromatic properties [9]. RM is a medicinal plant which is traditionally used in folk medicine for healing hyperglycemia, rheumatism, microbial infections, epilepsy, spasms, fluid retention, and fever [10].

Previous research has demonstrated that RM, particularly its EOs, exhibits a range of biological activities, including antimicrobial [11], antioxidant [12], antidiabetic [13], antitumor [14], antihypertensive [15], insecticidal, and larvicidal properties [16]. Despite the existing studies on RM, many of its biological and pharmacological activities remain unexplored.

In this regard, this exploratory study aimed to assess the phytochemical profile and the *in vitro* antiradical, antimicrobial, and antidiabetic activities of *Ruta montana* essential oil (RMEO) obtained from the Middle Atlas region of Morocco. Additionally, computational analyses were conducted to evaluate the pharmacokinetic parameters and drug-likeness of RMEO molecules, as well as to predict their toxicity profile. To the best of our knowledge, this is the first report investigating the *in vitro* antihyperglycemic potential of RMEO and its antioxidant activity using the β-carotene bleaching (BCB) assay. By highlighting these unexplored aspects, the present work addresses a key knowledge gap and provides novel insights into the therapeutic potential of RMEO.

## 2. Material and Methods

### 2.1. Plant Matter and Volatile Oil Isolation

The aerial portions of *Ruta montana* L. were collected from the Boulmane region. The plant was authenticated by the Botanical group at USMBA, and voucher samples were deposited in the botanical archive with corresponding ID codes (BLMUP-349). The volatile oil has been obtained through an adapted Clevenger-type device. Water was boiled with dried aerial parts (500 g) for 4 h. After carefully separating the collected EO, it was dried with anhydrous sodium sulfate (Na_2_SO_4_) to remove any residual water. Dried oils were preserved in an obscure environment at 4°C prior to assessment. The essential oil yield was calculated based on the dry weight of the plant material from three independent extractions. The yield of EO (% v/w) was calculated using this formula:

(1) $$\begin{array}{c} \text{Yield}\left(\%v/w\right)=\frac{\text{Volume of EO obtained}\left(\text{mL}\right)}{\text{Dry wright of plant material}\left(g\right)}\times 100. \end{array}$$

### 2.2. GC–MS Analysis

RMEO was chemically characterized by a GC–MS system with a BPX25 capillary column containing 95% of dimethylpolysiloxane diphenyl stage. The system used helium as the mobile phase gas, with a steady flow rate of 3 mL/min, and integrated a QP2010 mass detector (Kyoto, Japan). The mass spectra were studied within the area 40–300 *m*/*z*. Once the methanol and EO were mixed, 1 μL of the diluted oil was inserted in a split manner at a 90:1 ratio into the chamber. The injector temperature is at 20°C. The oven temperature program had an initial temperature of 60°C (held for 3 min), ramped at 3°C/min to 240°C, and then held for 10 min. Mass spectra were recorded in the 40–300 *m*/*z* range. To identify the constituents, retention times were compared with recognized standards, and mass spectra were matched against the NIST library. Retention indices (Kovats indices) were also calculated and used for confirmation of compound identities. Data processing was performed using Lab Solutions Software (Version 2.5).

### 2.3. Microbial Strains

The antibacterial activity of RMEO was assessed using five microbial strains. These included one fungus, *Candida albicans* (clinical isolate); Gram-negative (Gram−) bacteria, *Pseudomonas aeruginosa* ATCC 27853 and *Escherichia coli* ATCC 25922; and Gram-positive (Gram+) strains, *Micrococcus luteus* ATCC 14452 and *Staphylococcus aureus* ATCC 29213. Every strain was acquired from the Laboratory of LM2BM of FST. Bacteria were cultured on Luria–Bertani (LB) agar medium at 4°C. Prior to manipulation, yeast was resurrected by subculturing on Sabouraud agar (SA) plates at 25°C for 48 h, and bacterial strains were resurrected by subculturing in LB at 37°C for 20–24 h.

Antimicrobial screening was performed using final inoculum values of 10^6^ CFU/mL for bacteria and about 10^4^ CFU/mL for yeast [17].

### 2.4. Agar Disc-Diffusion Technique

According to Benkhaira et al. [18], the disc diffusion technique was first used for antibacterial screening. The culture suspension was applied to SA for yeast and LB agar for bacteria. After being soaked in 12 μL of pure EO, each sterile paper disc (6 mm) was put on the agar plate. The standard controls for yeast and bacterial strains were fluconazole (10 μg/disc) and kanamycin, respectively. Yeast plates were cultured for 48 h at 25°C, while bacterial plates were cultured at 37°C for 24 h. Millimeters were used to measure the inhibition zone widths, and the mean ± SD of three separate measurements was used to report the results.

### 2.5. Minimum Inhibitory Concentration (MIC) Assay

A previously established approach with slight changes was employed to assess the RMEO's MIC by Benkhaira et al. [18]. EO concentrations between 8.0% and 0.007% (v/v) were diluted in MH broth with 0.15% agar in sterile 96-well plates. Each well was filled with 10 μL of the produced bacterial suspension. The 96-well plates were cultured at 35°C overnight. Yeast was treated using the same procedure, which involved incubating it for 48 h at 25°C in a broth comprising peptone yeast extract. The standard growth control was MH broth without microbial suspension. Kanamycin was used as a reference antibiotic for both Gram-positive and Gram-negative bacteria. The concentrations were selected based on previously reported inhibitory ranges for the tested strains (CLSI guidelines and literature references). After the incubation period, 10 μL of resazurin, a measure of microbial growth, was added to each well. The color changed from blue-violet to pink after 2 h of incubation at 37°C, indicating the presence of microbiological growth. The lowest concentration at which resazurin does not change color is known as the MIC metric.

### 2.6. Identification of MBC and MFC Values

A volume of 5 μL from each well of the microplate that did not exhibit observable growth was subcultured on earlier prepared Petri dishes, including LB medium for bacterial cells and YPG for yeast, to precisely determine the minimum lethal concentrations to kill bacteria (MBC) and fungi (MFC). Following that, these plates were incubated under the proper conditions for every type of microorganism. Following incubation, the lowest concentration of CMBs and CMFs at which no microbial colonies were visible was identified. To further clarify the possible mode of action of the compounds under examination, the MBC/MIC and MFC/MIC values were measured [19].

### 2.7. *In Vitro* Assessment of α-Amylase Inhibition

With some modifications, the pancreatic α-amylase inhibition was computed using the procedure of El Hachlafi et al. [19]. The preincubation contained 200 μL of phosphated buffer (0.2 M; pH = 6.9), 200 μL of α-amylase solution (13 IU), and 100 μL of different concentrations of RMEO or acarbose (0.1–0.5 mg/mL). The preincubation was maintained at 37°C for 10 min. Immediately after preincubation, a 200 μL 1% starch mix (phosphate buffer solution) was combined with the reagent mixture. The reagent solution was kept for 20 min at 37°C and then stopped with 600 μL of 3.5 dinitrosalicylic acid (DNSA) color reagent. The tubes were subsequently kept in hot water at 100°C for 8 min and chilled in frozen water for 5 min. Then, the reagent suspension was diluted with distilled water down to 1 mL, and absorbance was determined at 540 nm. The assessments were realized thrice. Data were stated as means ± SD. The α-amylase inhibiting effect was computed through this equation:

(2) $$\begin{array}{c} \%\text{ Inhibition}=\frac{\left[\left(\text{Abs Control}-\text{Abs Samples}\right)\right]}{\text{Abs Control}}\times 100. \end{array}$$

### 2.8. Intestinal α-Glucosidase Inhibitory Effect *In Vitro*

The impact of the studied EO on the inhibition of the α-glucosidase enzyme was ascertained using the technique of Bouhrim et al. [20]. The solution was made in a final volume of 1220 μL by combining 100 μL of 50 mm sucrose, 100 μL of enzyme solution containing 10 IU of α-glucosidase, and 1000 μL of phosphate buffer at a concentration of 50 m and a pH of 7.5, plus different concentrations of EO or of acarbose solution (20 μL each). As a control, 10 μL of the phosphate buffer containing all of the reactants was used. Afterward, the mixtures were kept in heated water at 37°C for 25 min. The various solutions were then heated to 100°C for 5 minutes in order to terminate the enzymatic reaction. The D-glucose oxidase technique was used to determine the amount of D-glucose emitted. Each test was conducted with three replicates, with the results thus far reported as the mean ± SD. Absorbance was recorded at 500 nm, and the rate of inhibition was evaluated through the next equation:

(3) $$\begin{array}{c} \%\text{ Inhibition}=\frac{\left[\left(\text{Abs Control}-\text{Abs Samples}\right)\right]}{\text{Abs Control}}\times 100. \end{array}$$

### 2.9. Reductive Ferric Capacity Assay

The assessment of RMEO in terms of the ferric capacity was completed using a modified method developed by El Hachlafi et al. [21]. As a summary, RMEO was blended with 250 μL of potassium phosphate buffer and with 250 μL of 1% potassium ferrocyanide solution. The solution was kept at 48°C for 35 min. Afterward, 250 μL of 10% trichloroacetic acid was introduced. After centrifuging at 3500 rpm for 10 min, the supernatant was combined with 375 μL of hydrogen peroxide and 375 μL of 0.1% iron (III) chloride. The absorbance was compared to ascorbic acid, which served as a control. The readings were taken at 700 nm. The experiments were repeated thrice, and the outcomes were measured as EC_50_ ± SD.

### 2.10. BCB Assay

The BCB test was executed as designated by Kandsi et al. [22]. A mixture of β-carotene and linoleic acid emulsion was added to Tween-80. Afterward, a quantity of 2 mL was incorporated with 500 μL of different quantities of RMEO. The absorbance was recorded at 470 nm using methanol as blank. The optical density values were compared with references (BHT and ascorbic acid). Every experiment was conducted thrice.

### 2.11. Drug-Likeness, Absorption, Distribution, Metabolism, and Excretion (ADME) Properties, and Toxicity Prediction (Pro-Tox II)

ADME are the essential aspects that define the pharmacokinetic parameters of any substance in the biological context. Modeling tools are used to estimate the membrane permeation, as well as the activity of transporters and metabolizing enzymes responsible for drug intake and elimination and metabolic turnover. SwissADME and pkCSM, which are Webserver ADME databases, were used to evaluate the chemical and physical possessions, drug-likeness, and other pharmacokinetic characteristics of the constituents in the study [23, 24]. To determine the toxicity level, we used the Pro-Tox III digital platform (https://tox.charite.de/protox3/index.php?site=compound_input on August 11, 2024) [25], which assesses toxicity risk by placing the chemical structure into a statistical algorithm for known toxic chemicals.

This tool is used to predict the toxicity of the compound or ill effects upon its exposure to humans or other animals based on some data concerning LD_50_ points, toxicity level, and other adverse effects like hepatotoxicity, nephrotoxicity, mutagenicity, immunotoxicity, and cytotoxicity. The model and the devices ensure valuable information for potential therapeutic use and toxicity of RMEO chemicals.

### 2.12. Molecular Docking Protocol

The molecular docking experiments were performed to predict the interaction profile of the major phytoconstituents of RMEO with relevant molecular targets associated with antibacterial, antifungal, and antidiabetic activities [26].

#### 2.12.1. Ligand Preparation

The phytocompounds were retrieved from the PubChem database in SDF format using their respective CIDs and subsequently imported into *Discovery Studio* Version 4.5, where a ligand library was established. All ligands and reference compounds underwent geometry optimization using the PM6 semi-empirical method to minimize steric clashes and ensure accurate molecular conformations [27]. The optimized ligands were then saved in PDB format for docking.

#### 2.12.2. Protein Preparation

The selected target proteins were obtained from the RCSB Protein Data Bank on August 22, 2024, including dihydrofolate reductase (DHFR, PDB ID: 4M6J) for antibacterial activity [28], sterol 14-α-demethylase (CYP51, PDB ID: 1TZ1) for antifungal activity [26], α-glucosidase (PDB ID: 5NN5), and α-amylase (PDB ID: 1SMD) for antidiabetic activity [29]. Protein structures were prepared using *PyMOL* 2.3, where all crystallographic water molecules, cocrystallized ligands, and nonessential residues were removed. Nonpolar hydrogens were added to complete the valence structure, followed by energy minimization using *Swiss-PDB Viewer* [30]. The minimized proteins were exported in PDB format for subsequent docking.

#### 2.12.3. Docking Protocol

Molecular docking was carried out using AutoDock Vina 1.2.0, with the active site defined according to the coordinates of the native cocrystallized ligands. The grid box dimensions were adjusted to fully encompass the active site cavity (Table 1), ensuring optimal conformational flexibility of ligands. For each target, the grid center was defined as follows: DHFR (*x* = 7.545, *y* = 7.421, *z* = −19.225), CYP51 (*x* = 60.412, *y* = 50.521, *z* = 21.821), α-glucosidase (*x* = 1.591, *y* = −26.552, *z* = 87.364), and α-amylase (*x* = 8.349, *y* = 58.705, *z* = 19.096). The grid box sizes were uniformly set at 40 × 40 × 40 Å with a spacing of 1.0 Å, which adequately covered the binding pocket while preventing artifacts from peripheral binding.

#### 2.12.4. Validation of Docking Protocol

To ensure reliability, re-docking of the cocrystallized ligand for each protein was performed under the same docking parameters. The protocol was considered valid when the docked pose reproduced the crystallographic orientation of the native ligand with an root mean square deviation (RMSD) ≤ 2.0 Å. In our case, re-docking yielded an RMSD of below 2.0 Å (ranging from 0.57 to 1.69 Å), confirming the robustness and predictive accuracy of the docking workflow.

#### 2.12.5. Scoring and Binding Analysis

The docking scores, expressed as binding affinities in kcal/mol, were used to rank the phytoconstituents. Lower (more negative) binding affinity values corresponded to stronger binding interactions. The best docking poses were further analyzed for hydrogen bonds, hydrophobic interactions, and other noncovalent interactions using Discovery Studio Visualizer v 4.5.0.

### 2.13. Statistical Analysis

The trials were executed in triplicate, independently, and the findings reported here are calculated as mean ± SD. Statistical assessment was conducted with GraphPad 9, and comparisons were realized by the Tukey test (ANOVA).

## 3. Results and Discussion

### 3.1. Chemical Composition

Ten compounds have been revealed by GC–MS analysis, making up 97.92% of the entire composition. The properties of those detected compounds, including the molecular formulas, molecular weights, retention times, and relative peak areas, are presented in Table 2. The major part of these EO molecules belongs to the ketones class, constituting about 73.32% of RMEO. Among these, 2-undecanone is noted as the major constituent, with an amount of 56.41% in the oil, followed by 2-nonanone (8.92%), 2-undecanol (6.61%), and 2-decanone (5.21%). Monoterpene compounds, for example limonene (10.28%) and sabinene (4.10%), were also present in moderate concentrations (14.38%).

These findings are in corroboration with other studies that identified 2-undecanone as the major component in Moroccan RMEO. According to Benali et al. [32], 2-undecanone was found to constitute 63.97%. Likewise, chemical analysis of 11 samples of *R. montana* invariably identified 2-undecanone as the most prominent component [33]. This compound is often referred to as a common significant constituent of many samples collected from Algeria and Tunisia, which shows the chemical variability of this species. Concentration of 2-undecanone is variable, ranging anywhere from 20.9% to 81.7% [16, 34–36] depending on the particular location on the ground. Kambouche et al. [36] reported 2-undecanone at 32.8%, while Boutoumi et al. [16] described it at 67%–67.4% in RMEO. Notably, principal component analysis (PCA) used by Boutoumi et al. [16] to study RMEO from seven regions in Algeria noted a distinction of two groups: the one with a higher population of 2-undecanones and the one comprising 2-undecanone, 2-nonanone, and 2-nonanol-acetate. By contrast, Mohammedi et al. [37] found that RMEO from Tunisia displayed a quite different profile, with 1-butene as the major one, composing about 38.33% of the oil.

Overall, there can be drastic changes in the phytochemical profile of RMEO attributed to several determinants. First among these is the geographical origin of where the oil comes from; oils from different locations usually have separate profiles. Other factors that also affect composition include harvesting time, seasonal changes, environmental conditions like temperature and soil composition, and methods of extraction. Analytical techniques and varieties of the plant add further to these composition differences.  Table 3 shows the differences in the chemotypes of RMEO compared to previous studies. The data in the table highlights the variability observed across different regions and emphasizes the chemical diversity of the species.

### 3.2. Antimicrobial Activity

There were significant differences in the antibacterial activity of RMEO on the test microbes listed in Tables 3 and 4. Of the studied microbes, *M. luteus* showed the highest susceptibility with the maximum inhibition zone size of 20.05 ± 0.98 mm, followed by *P. aeruginosa*, which showed minimum resistance with an inhibition zone of 15.11 ± 0.75 mm. Furthermore, RMEO has moderate anticandidal activity against *C. albicans* (13.05 ± 0.5 mm) (Table 4). The microdilution test supported the disk diffusion results, revealing low MIC metrics were observed from 2.25 to 36 mg/mL toward the investigated microbial species. Furthermore, the MBC/MIC and MFC/MIC proportions showed that RMEO had a bactericidal/fungicidal impact on all strains (Table 5). The antibacterial impact of RMEO was comparable to those of the reference medicines kanamycin and fluconazole.

The observed variation in susceptibility between Gram-positive and Gram-negative bacteria can be rationalized by differences in cell wall architecture. Gram-positive bacteria, such as *M. luteus*, lack the outer membrane present in Gram-negative bacteria, facilitating the penetration of hydrophobic essential oil constituents and leading to higher antimicrobial efficacy. In contrast, the outer membrane of Gram-negative bacteria, such as *P. aeruginosa*, acts as a barrier, reducing the effectiveness of EOs [37].

Chemical analysis of RMEO revealed the presence of major compounds, including 2-undecanone and 2-undecanol, which have been previously reported to exhibit significant antimicrobial activity [44]. The lipophilic nature of these compounds likely enables their integration into microbial cell membranes, causing disruption of membrane integrity, leakage of cellular contents, and eventual cell death. Thus, the antimicrobial activity of RMEO can be attributed, at least in part, to these key constituents.

Most previous studies on RM have reported a variety of antimicrobial mechanisms of action. Undercurrent tests, the moderate antimicrobial effect of RMEO against a large variety of nosocomial strains was shown by Flouchi et al. [40]. Among these singled-out bacteria are Gram-positive (*S. aureus*) and Gram-negative (*Pantoea* spp., *P. aeruginosa*, *Klebsiella pneumoniae*, *E. coli*, *Escherichia hermannii*, and *Stenotrophomonas maltophilia*). Furthermore, Benali et al. [32] found that RMEO had strong anticandidal action against *C. albicans*. The convergence of chemical composition and observed bioactivity suggests a plausible structure-activity relationship, where the presence of medium-chain ketones and alcohols enhances the ability of RMEO to penetrate microbial membranes [40].

Previous research has shown that RMEO exhibits greater efficacy against Gram-positive than Gram-negative bacteria. One such reason could be the increased permeability of any strain's wall to hydrophobic chemicals (EOs), resulting in cell activity. However, Gram-negative bacteria have an outer membrane that is more resistant to EOs [45]. Furthermore, the presence of 2-undecanone and 2-undecanol, two key components of the oil with antimicrobial activity, may explain RMEO's antibacterial action [44].

Taken together, these findings highlight the potential of RMEO as a natural antimicrobial agent, particularly against Gram-positive bacteria and opportunistic fungal pathogens. Its comparable efficacy to standard antibiotics underscores its relevance for developing alternative or adjunctive antimicrobial strategies.

### 3.3. Suppressive Activity Against α-Amylase and α-Glucosidase Enzymes

**Figure 1:**
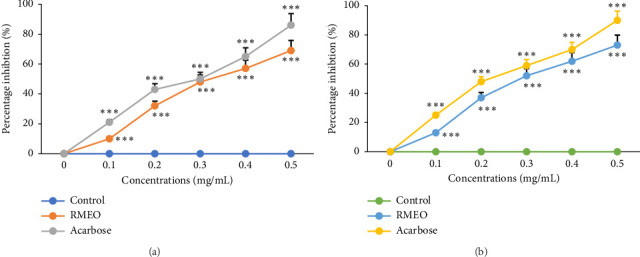
Inhibitory effect of α-amylase (a) and α-glucosidase (b) enzymes by RMEO and acarbose *in vitro.* The values are the means ± SEM (*n* = 3). ^∗∗^*p* < 0.01, ^∗∗∗^*p* < 0.001 as function of acarbose.

As stated by Figure 1, in a concentration-responsive effect (*p* < 0.001), RMEO demonstrated strong restriction of α-amylase and α-glucosidase enzymes, with IC_50_ metrics of 0.455 ± 0.11 and 0.401 ± 0.04 mg/mL, respectively (Table 6). The suppression activity of RMEO against α-amylase and α-glucosidase enzymes was somewhat closer to those recorded for acarbose (IC_50_ = 0.281–0.401 mg/mL).

Although no previous studies have directly assessed the *in vitro* antihyperglycemic activity of RMEO, related species provide useful context. Loizzo et al. [46] reported that methanolic extracts of *Ruta chalepensis* leaves inhibited α-amylase and α-glucosidase with IC_50_ values of 69.0 and 85.5 μg/mL, respectively, while an aqueous extract of RM improved glucose tolerance in hyperglycemic rodents and prevented increases in blood sugar levels [13]. These studies collectively support the idea that *Ruta* species contain bioactive compounds capable of modulating carbohydrate metabolism.

The pronounced inhibitory effect of RMEO on α-amylase and α-glucosidase may be attributed to its major chemical constituents, such as 2-undecanone and 2-undecanol, which are lipophilic and could interact with the active sites of these enzymes. This is consistent with structure-activity principles, where the chemical structure allows tight binding to the enzyme catalytic sites, leading to enzyme inactivation [13].

Moreover, molecular docking studies on essential oil constituents have suggested that these molecules can interact with the binding pockets of α-amylase and α-glucosidase, effectively reducing their activity. By inhibiting these key digestive enzymes, RMEO may reduce carbohydrate hydrolysis, limit intestinal glucose absorption, and consequently lower postprandial blood glucose levels [47].

Taken together, these findings highlight the potential of RMEO as a complementary natural strategy for managing hyperglycemia, with efficacy approaching that of acarbose. Further studies, including *in vivo* evaluations and isolation of active constituents, are warranted to fully elucidate the mechanisms underlying its antidiabetic effects.

### 3.4. Antioxidant Activity

EOs have attracted significant interest due to their diverse therapeutic benefits, particularly their antioxidant potential [48, 49]. The quest for natural antioxidants has driven research toward these complex mixtures of phytochemicals, which include terpenes, ketones, esters, and phenolics. Antioxidants neutralize free radicals and reactive oxygen species, which help to alleviate oxidative stress linked to many chronic diseases and aging [50, 51]. Indeed, it was recently established that EOs can inactivate free radicals, decreasing oxidative stress and potentially protecting against multiple diseases [52]. By mitigating oxidative stress, antioxidants can protect cellular macromolecules from damage, thereby potentially preventing the onset or progression of disorders such as diabetes, cardiovascular diseases, and cancer.

**Figure 2:**
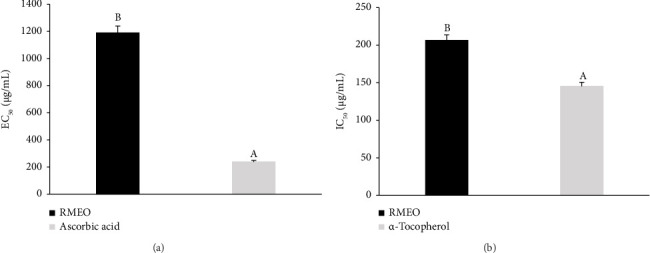
Antioxidant potential of RMEO, (a) EC_50_ of RP test, (b) IC_50_ of BCB assay. Data with different letters in the same assay indicate significant differences (Tukey's multiple range test, ANOVA, *p* < 0.05); identical letters indicate no significant difference.

The antioxidants in RMEO were tested using two *in vitro* assays: ferric reducing power (RP) and BCB. The findings were compared with some classical antioxidant substances, such as ascorbic acid and α-tocopherol, in order to show the complete potential of RMEO, its efficacy, and its application for future use (Figure 2). RMEO demonstrated effective lipid peroxidation inhibition in the BCB assay (IC_50_ = 206.21 ± 1.12 μg/mL) and noticeable reductive power in the RP test (EC_50_ = 1188.61 ± 3.49 μg/mL), indicating its capacity to donate electrons and neutralize free radicals, although its efficacy is lower than that of standard antioxidants.

The antioxidant activity of RMEO can be attributed to its major chemical constituents, such as 2-undecanone, 2-undecanol, and other phenolic or oxygenated terpenes, which are known to possess radical scavenging and electron-donating properties. The variability in activity reported in previous studies [11, 32, 37] further suggests that the composition of RMEO, and thus its antioxidant potential, is highly dependent on geographical origin, environmental conditions, and harvesting practices. This emphasizes the importance of chemical profiling to correlate specific constituents with observed bioactivity.

Notably, this study is presumably the first to report RMEO's activity in the BCB assay, highlighting its potential to prevent lipid peroxidation. Mechanistically, the phenolic and oxygenated components of RMEO can donate hydrogen atoms or electrons to free radicals, stabilizing them and interrupting radical-mediated chain reactions. This provides a molecular basis for its protective effect against oxidative stress.

Overall, the antioxidant potential of RMEO represents an intersection of traditional knowledge and modern scientific validation. By elucidating the chemical composition and mechanisms underlying its activity, RMEO could serve as a natural source of antioxidants for therapeutic applications, functional foods, and the cosmetic industry. Its ability to modulate oxidative stress also suggests potential benefits in managing disorders related to oxidative damage, such as tumors, diabetes, and cardiovascular diseases [32].

### 3.5. Computational Analysis of the Pharmacokinetic Profiles (ADME) of RMEO Compounds

*In silico* drug-likeness assessment has become an integral part of drug discovery strategies, whereby the filtration and prioritization of the most promising candidates may be accomplished through less exigent processes [53, 54]. The *in silico* practices optimize both resources and time, alleviating tentative burden and allowing for the rapid identification of molecules with desirable pharmacokinetic properties and target interactions [55, 56]. Lipinski's rule of five requires particular physicochemical properties (fewer than 5 H-bond donors, fewer than 10 H-bond acceptors, no more than 10 N or O atoms, molecular weight less than 500 Da, and MLOGP less than or equal to 4.15). All the n-derivatives meet Lipinski's rule of five, which is really interesting. Furthermore, all of the compounds found in RMEO satisfied other drug-likeness standards (Veber's and Egan's) (Table 7). According to Martin's find resolution obtained in 2005, compounds that followed Lipinski's rule of five shall be credited with a bioavailability index of 0.55. Consequently, all of the recognized organic composites can be attributed a bioavailability index of 0.55 [57].

**Figure 3:**
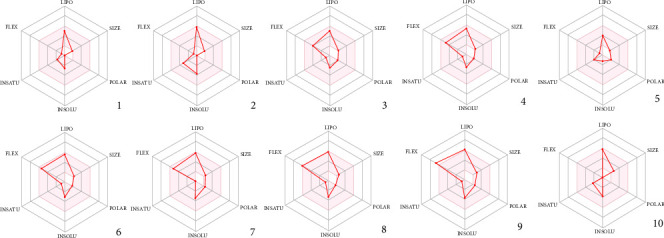
Bioavailability radars of the volatile composition of RMEO. (1) Sabinene, (2) limonene, (3) 2-nonanone, (4) 2-decanone, (5) 5-norbornene-2-methanol, (6) 2-undecanone, (7) 2-undecanol, (8) 2-dodecanone, (9) 2-undecanol acetate, and (10) caryophyllene.

The bioavailability assessments of the volatile constituents in RMEO, as depicted in Figure 3, demonstrate the potential suitability of these phytochemicals as drug candidates. Each radar plot represents the bioavailability radar, a graphical tool designed to evaluate whether a molecule falls within the optimal chemical space for oral bioavailability. The pink region corresponds to the ideal physicochemical domain where drug-like molecules are most likely to show good gastrointestinal absorption and oral bioavailability. A compound is considered to meet drug-likeness criteria if its red polygon is entirely contained within this pink zone.

Each axis of the radar represents a key molecular descriptor:

• LIPO (lipophilicity): Optimal partition coefficient (XLOGP3 between −0.7 and + 5.0) ensures balanced solubility and permeability.
• SIZE: Refers to molecular weight (150–500 g/mol), as excessively large molecules may have poor absorption.
• POLAR: Polar surface area (20–130 Å^2^), which influences hydrogen bonding capacity and passive diffusion.
• INSOLU (insolubility): Indicates solubility in water (log S not higher than 6), essential for dissolution in the gastrointestinal tract.
• INSATU (unsaturation): Fraction of carbons in sp^3^ hybridization (> 0.25 desirable), linked to structural complexity and metabolic stability.
• FLEX (flexibility): Number of rotatable bonds (≤ 9 preferred), as highly flexible molecules may have reduced binding specificity and permeability.

The radar plots of the investigated compounds, sabinene, limonene, 2-nonanone, 2-decanone, 5-norbornene-2-methanol, 2-undecanone, 2-undecanol, 2-dodecanone, 2-undecanol acetate, and caryophyllene, show that these constituents largely fall within the drug-like physicochemical space. This compliance with oral bioavailability criteria suggests that these volatile compounds possess favorable pharmacokinetic characteristics, reinforcing their potential as promising lead molecules for therapeutic applications.

A medicine may lose effectiveness due to poor ADME possessions. Additionally, the major obstacle faced by drug discovery throughout the clinical investigations is the pharmacokinetic properties of the drug in the clinical stages. That could run costs sky-high. Hence, *in silico* techniques were applied to evaluate the ADME characteristics so as to estimate the ability of RMEO as a drug development candidate. A number of aspects were considered in this study, such as physical and chemical properties, along with aspects of absorption, distribution, metabolism, and excretion.

With respect to the logS scale, compounds exhibiting solubility in the range of −4 to 0 are characterized by good solubilization [23]. Thus, in this regard, apart from compounds like 2-nonanone, 2-decanone, and 2-dodecanone, the rest are said to have favorable water solubility. All the phytochemical compounds exhibit significant Caco-2 permeability, as indicated by their log *p* values in the range of 10−6 cm/s (Table 8) [58]. Consequently, they demonstrate a substantial percentage rate in the human intestine, spanning from 88.94% to 95.97%. Log *K*_*p*_ (in cm/s) serves as a crucial indicator of a molecule's ability to permeate the skin, particularly in relation to transdermal drug delivery [24]. Generally, a little skin permeability is validated when a log *K*_*p*_ value of a molecule is in excess of −2.5 cm/s; none of the compounds studied in this work exceeded this log *K*_*p*_ value, indicating thus a limited capacity for skin permeability (Table 9).

Dss, the steady-state volume of distribution, may be defined as the theoretical volume in which the whole bulk of a drug or molecule is uniformly dispersed so as to give a concentration equal to that found in plasma [59]. Typically, VDss is said to be amplified when it is above 2.81 L/kg, or when log VDss is greater than 0.45 [24]. If, however, it is lower than 0.71 L/kg or the log VDss is less than −0.15, it is considered low [24]. Based on their VDss results, which range from 0.206 to 0.652, these compounds exhibit moderate distribution capacities. The blood–brain barrier (BBB) plays a crucial role in protecting the brain from exogenous molecules. An important consideration in this platform is the potential of a substance to cross the BBB. Lower levels of this parameter mean lower prospects for toxicities and side effect inductions while improving efficacy in a drug or molecule for which the desired pharmacological action lies somewhere within the brain. Log BB values above 0.30 imply a high possibility of distribution into the brain and indicate that these compounds have the potential to hit various neurological targets and activities.

The detoxification enzyme cytochrome P450 plays a pivotal role and is localized in the largest part of the liver [60], while discussing metabolism parameters. The compounds that inhibit this enzyme could significantly interfere with drug metabolism, which is commonly discouraged because of possible adverse effects. None of the constituents were predicted to be inhibitors or substrates of the CYP450 enzymes with a primary focus on isoforms CYP2D6 and CYP3A4. Inference could therefore be made that these compounds assumed low chances of interfering with drug metabolism, which strengthens the security of these molecules.

The overall clearance rates for the composites span from 0.071 to 1.636, revealing variable rates of elimination. No substances were found as renal OCT2 substrates, suggesting that these compounds might have a reduced risk of renal toxicity.

**Figure 4:**
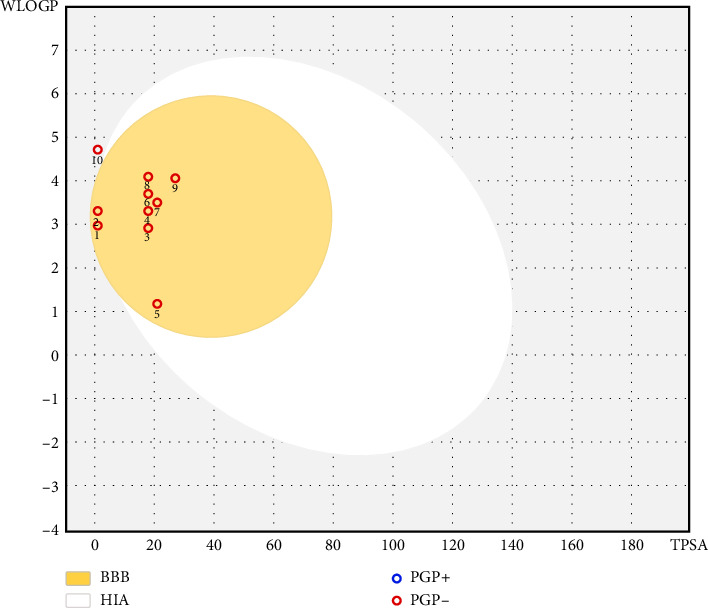
Boiled-egg model of the BBB permeability and GI absorption of the major compounds in CAEO. (1) Sabinene, (2) limonene, (3) 2-nonanone, (4) 2-decanone, (5) 5-norbornene-2-methanol, (6) 2-undecanone, (7) 2-undecanol, (8) 2-dodecanone, (9) 2-undecanol acetate, and (10) caryophyllene.

The BOILED-Egg model (Figure 4) is a widely used predictive tool for assessing two crucial pharmacokinetic properties of candidate molecules: BBB penetration and human intestinal absorption (HIA). The model is based on lipophilicity (WLOGP) and polarity (TPSA), which are two key molecular descriptors influencing absorption and distribution [61]. In this visualization, the white ellipse represents the physicochemical space associated with high gastrointestinal absorption, while the yellow “yolk” denotes molecules with the potential to permeate the BBB. In addition, color coding indicates whether molecules are substrates of the P-glycoprotein (P-gp) efflux transporter, which influences central nervous system (CNS) drug delivery: blue dots represent P-gp substrates, and red dots represent nonsubstrates.

Our results show that almost all identified phytochemicals from RMEO fall within both the white region (high GI absorption) and the yellow yolk (high BBB permeability), indicating excellent predicted oral absorption and strong potential for CNS activity. Importantly, the majority of these compounds are predicted not to be P-gp substrates (red dots), suggesting they are less likely to be actively effluxed from the brain, which enhances their CNS bioavailability. Caryophyllene was the only compound positioned differently, showing a distinct profile compared to the others.

Taken together, these findings provide strong evidence that the volatile constituents of RMEO not only have favorable oral bioavailability but also possess the physicochemical characteristics needed to cross the BBB. This highlights their therapeutic promise, particularly for conditions involving the CNS.

### 3.6. Computational Prediction of Organ Toxicity and Toxicological Outcomes

*In silico* toxicity analysis of RMEO would imply that the majority of the major compounds may have either moderate or very high toxicities depending on endpoints, which include hepatotoxicity, nephrotoxicity, carcinogenicity, immunotoxicity, mutagenicity, and cytotoxicity [62–64]. Most compounds, such as sabinene, limonene, 2-nonanone, and 2-decanone, are predicted as ineffective, thus posing low risk for potential toxicity. For example, sabinene and limonene are predicted to exhibit significant probabilities of being inactive in nephrotoxicity (0.91 and 0.85, respectively) and in immunotoxicity (0.82 and 0.95, respectively); the estimated LD_50_ for sabinene and limonene-V being 5000 mg/kg and 4400 mg/kg shows these compounds are likely to be harmful if swallowed but less toxic [65]. Also, 2-nonanone and 2-decanone are inactive in all endpoints with predicted LD_50_s of 5000 mg/kg and 4300 mg/kg, respectively, putting them in Class V. On the other hand, 2-undecanone is inactive, but with a predicted LD_50_, it would be placed in Class IV (evaluation with the value 1000 mg/kg), indicating comparatively higher toxicity risk. Curiously, caryophyllene stands out with a predicted LD_50_ value of 5300 mg/kg, which places it in Class VI, indicating it is not harmful when swallowed, hence indicating it is the least toxic among the various compounds compared in this evaluation. While the ADME profiling suggests that most RMEO constituents display favorable drug-likeness and safety features, the predicted toxicity profile of 2-undecanone warrants more cautious interpretation. Notably, 2-undecanone was classified as a Class IV compound based on its LD50, indicating a comparatively higher toxicity risk relative to other constituents such as sabinene, limonene, and caryophyllene. This finding is particularly relevant given that 2-undecanone constitutes the major fraction of RMEO (56.41%). Although its bioavailability, absorption, and metabolic stability parameters are favorable, its elevated toxicity score underscores the need for careful dose optimization and further *in vitro* and *in vivo* validation before therapeutic applications can be considered. Importantly, the predicted toxicity does not preclude its potential pharmacological utility but highlights the necessity of balancing efficacy with safety in the context of RMEO's use. Moreover, the overall safety profile of the oil may be influenced by synergistic or antagonistic interactions among its constituents, which could attenuate or amplify the toxic potential of 2-undecanone. Future experimental toxicological studies should therefore focus on dose–response evaluations of 2-undecanone, both individually and within the complex phytochemical matrix of RMEO, to better delineate its contribution to the oil's pharmacological activities and safety margins.

### 3.7. Molecular Docking Analysis of Potential Mechanisms of Action of RMEO Constituents

To elucidate the pharmacological actions of RMEO, molecular docking of its bioactive constituents with the corresponding molecular receptors was conducted using several *in silico* approaches. In docking studies, the binding strength of ligand-receptor interactions is inversely proportional to the binding affinity, expressed in kcal/mol. For each docking run, the grid box was carefully defined to encompass the active site of the receptor, ensuring accurate ligand placement. Validation of the docking protocol was performed through re-docking of the cocrystallized ligand into the active site, which yielded a RMSD of 0.0 Å, thereby confirming the reliability of the docking procedure. Under these validated conditions, the RX docking provided the most favorable predicted binding affinity using approach in [53].

This study evaluated the binding affinities of 10 molecules in RMEO against five proteins with diverse roles in biological systems: dihydrofolate reductase (DHFR, PDB ID: 4M6J) for antibacterial potential, cytochrome P450 alpha-sterol demethylase (PDB ID: 5TZ1), and two antidiabetic proteins, α-amylase (PDB ID: 1SMD) and α-glucosidase (PDB ID: 5NN5). The outputs of the *in silico* tests are displayed in a heat map (Table 10), with colors representing a gradient from red to blue, and white indicating the 50^th^ percentile to mark the energies of the docking outcomes. The values with the lowest energy, generally matching the native ligand or a highly active inhibitor, are presented in red, indicating the best matches. The blue shading or other varieties present would represent increased energy levels and reflect lower affinity toward the target. Such a procedure simplifies the identification of active compounds that show inhibition on a specific target.

The molecular docking study on the bioactive components of RMEO provides very promising information regarding expected pharmacological activities. The table shows these values in kcal/mol, which imply binding affinities of these compounds toward various molecular receptors that are connected with antibacterial, anticandidal, and antidiabetic activities. Lower binding affinity values are indicative of stronger binding and higher inhibition potential. The results are presented in gradient colors in shaded representation, with red for strong binding (i.e., low-energy scores), while blue indicates weak binding (i.e., high-energy scores).

Dihydrofolate reductase (DHFR) is one of the enzymes critical for tetrahydrofolate biosynthesis, which is important in the manufacture of pyrimidines, purines, and others [66]. Inhibiting DHFR has been a prominent site in antimicrobial, antituberculosis, and antitumor therapeutic research, while interrupting this pathway would hamper cells from being able to replicate or grow [67]. The protein crystal configuration was acquired from the RCSB Protein Databank with a PDB ID of 4M6J and represents the crystallographic structure of DHFR isolated from *S. aureus* [68]. This structural component shows as a promising alternative for antibacterial drug discovery since *S. aureus* is a highly virulent pathogen causing infections ranging from dermis infections to other ailments [68]. The focus on DHFR is also strategically significant since its inhibition will block the synthesis of tetrahydrofolate, thus leading to the diminution of the main folate coenzymes, which are critical for DNA, RNA, and protein synthesis. Therefore, it does state promising prospects for antibacterial drug development by inhibiting DHFR and controlling microbial growth and expansion [69].

So DHFR becomes a great target for antibacterial drug development, as it can inhibit bacterial multiplication and growth [70]. In this investigation, caryophyllene and ciprofloxacin (inhibitors used in this experiment) have the strongest binding affinity, both having a binding energy of −6.8 kcal/mol. Because of their strong binding, they can block the dihydrofolate reductase enzyme and serve as effective antibacterial agents. Other molecules, such as sabinene, limonene, 2-nonanone, and 2-decanone, show moderate binding affinity (−5.9 to −4.3 kcal/mol) with different degrees of antibacterial activity.

In terms of anticandidal activity, CYP51 encoded by the *Erg11* gene is an important member of the cytochrome P450 monooxygenase (CYP) superfamily. This enzyme is engaged in the manufacture of ergosterol, a sterol found exclusively in fungi [71]. Ergosterol is the primary sterol present in fungal cell membranes and is required to maintain membrane features such as structural integrity, fluidity, and permeability [72]. Ergosterol is also important for the proper functioning of membrane-bound enzymes, thus its importance in fungal cell physiology. For this purpose, the protein chosen was sterol 14-alpha demethylase (CYP51) from *C. albicans*, PDB ID: 5TZ1 [26]. The docking results showed that caryophyllene and VT1161 (Oteseconazole) [26] had the strongest binding affinities at −7.2 kcal/mol. This means they may be effective in inhibiting the cytochrome P450 alpha-sterol demethylase enzyme, a target for antifungal therapy. Sabinene, limonene, and 2-nonanone had moderate to weak binding affinities with metrics spanning from −5.8 to −4.4 kcal/mol and still have antifungal activity.

The major function of α-glucosidase is to hydrolyze polysaccharides into glucose by cleaving (β1–4) bonds. This increases the availability of glucose for absorption into the blood, therefore increasing blood glucose levels [73, 74]. The physiological effect of α-glucosidase consumes dietary starch and carbohydrates, delaying increases in blood glucose levels [75]. This enzyme catalyzes the absorption of monosaccharides in blood glucose and other carbohydrates from the intestine, which in turn would lead to high-level glucose concentrations in the blood [76]. α-Amylase functions as an enzyme in the cleavage of α-linked polysaccharides, forming α-anomeric isomers. It is integral to carbohydrate digestion and is present in saliva and pancreatic juice [77]. Three catalytic residues reside at the active site of α-amylase (PDB ID: 1SMD): Asp197, Glu233, and Asp300. Other residues that are known to modify the effect of α-amylase include Arg337, Arg195, Asn298, Phe265, Phe295, His201, Ala307, Gly306, Trp203, Trp284, Trp59, Tyr62, Trp58, His299, and His101 [75, 78].

The inhibitor acarbose holds the highest binding affinities as revealed in molecular docking studies against two targets (PDB IDs: 1SMD and 5NN5) in antidiabetic activity of −7.8 and −7.4 kcal/mol, respectively. The inhibitor caryophyllene also shows promising results with binding energies of −6.8 and −7.4 kcal/mol, suggesting that it can inhibit enzymes like α-amylase and α-glucosidase in which diabetic management is concerned. Other compounds, which are sabinene and 2-undecanone, only show moderate values of binding affinities and can be taken up for development into antidiabetic treatments but are less effective compared to caryophyllene and acarbose.

Caryophyllene thereby becomes significant as a potential compound with strong binding affinities against all tested targets, indicating a broad-spectrum pharmacological potential. Sabinene has shown moderate binding across different targets with varied pharmacological activity. Compounds such as limonene and 2-nonanone with rather lower affinities suggest little promise of being inhibitors. 2-decanone shows variable binding affinities, lower for antidiabetic targets but moderate for antibacterial and anticandidal activities. Compounds such as 2-undecanol, along with 2-undecanol acetate, propose moderate potential medicinal use under specific circumstances.

In addition to binding affinity values, the docking poses were analyzed to identify key amino acid residues involved in ligand recognition. For DHFR (PDB ID: 4M6J), caryophyllene and ciprofloxacin formed hydrophobic interactions with residues Ile5, Phe92, and Leu20, which are critical for stabilizing inhibitors within the folate-binding pocket. Caryophyllene also engaged in van der Waals interactions with Ala9 and Val115, contributing to its stable binding mode. These residues have been previously reported as essential for ligand accommodation and catalytic activity in DHFR inhibitors [68]. In contrast, sabinene displayed only weak hydrophobic contacts with peripheral residues, which is consistent with its lower binding affinity.

For CYP51 (PDB ID: 5TZ1), caryophyllene demonstrated strong hydrophobic interactions with residues Phe233 and Tyr132 located in the heme-binding channel while forming stabilizing van der Waals interactions with Met508 and His377. Such interactions have been reported to be crucial for the antifungal activity of azole inhibitors, which also target the heme catalytic site of CYP51 [26, 79]. These observations suggest that caryophyllene may mimic the binding mode of VT1161 (oteseconazole) and interfere with ergosterol biosynthesis.

Regarding α-amylase (PDB ID: 1SMD), acarbose established multiple hydrogen bonds with the catalytic residues Asp197, Glu233, and Asp300, as described in crystallographic studies [75]. Caryophyllene, although lacking strong hydrogen bonding, was stabilized by hydrophobic contacts with Trp59, Tyr62, and Phe295, residues known to modulate substrate accommodation [77].

In α-glucosidase (PDB ID: 5NN5), caryophyllene interacted with Phe157, Asp349, and Trp406 through hydrophobic interactions, indicating a potential inhibitory mechanism despite the absence of direct H-bonding. These residues have been implicated in stabilizing inhibitors at the catalytic site in recent docking studies [80].

Collectively, these interaction profiles demonstrate that hydrophobic stabilization within the catalytic pockets underlies the broad-spectrum binding potential of caryophyllene, while the lack of strong residue-specific contacts explains the moderate affinities observed for sabinene, limonene, and other minor constituents. The docking results put forward the conclusion that caryophyllene and the known inhibitor are two most likely candidates from RMEO acting mainly against the antibacterial, anticandidal, and antidiabetic targets. Other chemicals, for example, sabinene or 2-undecanol, have been determined as beneficial, but slightly farther from these goals. Such proposals could only be considered landmarks toward further experimental validation and development of RMEO-based therapeutic agents.

## 4. Conclusion

This systemic study analyzes the phytochemical profile and bioactivities of RMEO through *in vitro* and computational analysis. With strong antidiabetic, antimicrobial, and antioxidant activity, RMEO behaves beneficially. The bioactive compounds characterized in this oil are responsible, according to GC–MS analysis. RMEO has significantly inhibited α-amylase and α-glucosidase enzymes, marked strong antioxidant activity through ferric decreasing potential and BCB techniques, and significant antibacterial activity, especially against *M. luteus* and *S. aureus*, and good antifungal activity against *C. albicans*. The MIC, MBC, and MFC values attest to its ability to kill microorganisms at low concentrations. In addition to that, molecular docking of bioactive components of RMEO helps further understand the possible biological activities. Because of these promising results, further *in vivo* studies are valuable for determining the potency and safety of the oil in in vivo models. In addition, the complex interplay of essential oil compounds with various protein targets could provide significant agents for further exploration and advancement in therapies.

## Figures and Tables

**Table 1:** Molecular modeling targets and grid box characteristics.

Proteins/PDB IDs	Grid box size	Grid box center	Selected inhibitor	Results of docking validation with the cocrystallized ligand
Dihydrofolate reductase/4M6J	size_*x* = 40	center_*x* = 7.545	Ciprofloxacin	0.84 Å
	size_*y* = 40	center_*y* = 7.421		
	size_*z* = 40	center_*z* = −19.225		

Sterol 14-alpha demethylase (CYP51) from *Candida albicans*/5TZ1	size_*x* = 40	center_*x* = 60.412	Oteseconazole (VT-1161)	1.37 Å
	size_*y* = 40	center_*y* = 50.521		
	size_*z* = 40	center_*z* = 21.821		

α-Amylase/1SMD	size_*x* = 40	center_*x* = 8.349	Acarbose	0.57 Å
	size_*y* = 40	center_*y* = 58.705		
	size_*z* = 40	center_*z* = 19.096		

α-Glucosidase/5NN5	size_*x* = 40	center_*x* = 1.591	Acarbose	1.69 Å
	size_*y* = 40	center_*y* = −26.552		
	size_*z* = 40	center_*z* = 87.364		

^∗^RMSD value of the cocrystallized ligand is considered good when it is < 2.5 Å [31].

**Table 2:** Chemical composition of RMEO.

No.	Chemical compounds	Formula	Molecular weight (g/mol)	Retention time (min)	% Peak relative area
1	Sabinene	C_10_H_16_	136	9.068	4.10
2	Limonene	C_10_H_16_	136.24	10.754	10.28
3	2-Nonanone	C_9_H_18_O	142.24	12.556	8.92
4	2-Decanone	C_10_H_20_O	156.26	15.554	5.21
5	5-Norbornene-2-methanol	C_8_H_12_O	124.18	17.987	1.60
6	2-Undecanone	C_11_H_22_O	170.29	18.623	**56.41**
7	2-Undecanol	C_11_H_24_O	172.31	18.743	6.61
8	2-Dodecanone	C_12_H_24_O	184.32	21.127	1.25
9	2-Undecanol acetate	C_13_H_26_O	214.34	21.939	2.67
10	Caryophyllene	C_15_H_24_	204.36	22.036	0.94

Yield (%v/w)					3.21 ± 0.87

Total identified					**97.92**
Ketones					**73.32**
Monoterpene hydrocarbons					14.38
Alcohol					9.28
Sesquiterpene hydrocarbons					0.94

*Note:* The bold values represent the main compounds of the oil and the principal chemical groups of the volatile constituents.

**Table 3:** Major constituents of *Ruta montana* essential oils across recent studies.

Plant part used	Extraction method	Region of collection	Main compounds	Reference
Aerial part	Hydrodistillation	Algeria	2-Undecanone, octadecanol	[38]
Aerial part	Hydrodistillation	Middle Atlas, Morocco	2-Undecanone, decyl propanoate	[39]
Aerial part	Hydrodistillation	Taza region, Morocco	2-Undecanone, 2-nonanone, 2-decanone	[40]
Leaves	Hydrodistillation	Northeastern Algeria	2-Undecanone, 2-nonanone, 2-decanone	[41]
Leaves and stems	Hydrodistillation	Tunisia	2-Undecanone 1-nonene, 2-nonanone	[42]
Leaves	Hydrodistillation	Sfax, Tunisia	1-Butene, methylcyclopropane, 2-butene, caryophyllene oxide	[43]

**Table 4:** Evaluation of RMEO antimicrobial activity using the disc-diffusion test.

Microorganisms	Mean zone of inhibition (mm ± SD)		
	RMEO (10 μL/disc)	Kanamycin (15 μg/disc)	Fluconazole (15 μg/disc)
*Staphylococcus aureus* ATCC 29213	18.11 ± 1.11^b^	20.23 ± 0.15^a^	NT
*Micrococcus luteus* ATCC 14452	20.05 ± 0.98^b^	21.89 ± 0.21^a^	NT
*Escherichia coli* ATCC 25922	16.52 ± 0.08^b^	18.5 ± 0.01^a^	NT
*Pseudomonas aeruginosa* ATCC 27853	15.11 ± 0.75^b^	17.35 ± 0.11^a^	NT
*Candida albicans* (clinical isolate)	13.05 ± 0.5^b^	NT	15.02 ± 0.05^a^

*Note:* RMEO was applied at volumes expressed in μL/disc, whereas the standard antibiotics were applied at masses expressed in μg/disc. Within each microorganism row, means followed by different superscript letters (a, b) are significantly different at *p* < 0.05, according to Tukey's multiple range test. Identical letters indicate no significant difference.

Abbreviations: NT = not tested, SD = standard deviation.

**Table 5:** MIC, MBC, and MFC values of RMEO expressed in mg/mL.

Bacteria	RMEO			Kanamycin		
	MIC	MBC	MBC/MIC	MIC	MBC	MBC/MIC
*S. aureus*	4.5	18	36	2.25	9	36
*M. luteus*	2.25	9	36	4.5	9	4.5
*E. coli*	18	36	18	9	18	18
*P. aeruginosa*	36	72	18	9	18	18

**Fungi**	**RMEO**			**Fluconazole**		
	**MIC**	**MFC**	**MFC/MIC**	**MIC**	**MFC**	**MFC/MIC**

*C. albicans*	36	36	9	9	9	9

**Table 6:** IC_50_ values of RMEO and acarbose on pancreatic α-amylase and intestinal α-glucosidase enzymes.

	IC_50_ (mg/mL)	
	α-Amylase	α-Glucosidase
RMEO	0.451 ± 0.11^a^	0.401 ± 0.04^a^
Acarbose	0.306 ± 0.03^b^	0.281 ± 0.02^b^

*Note:* Values are expressed as mean ± SEM (*n* = 3). Data sharing the same letter within the same test indicates no significant difference (*p* < 0.05).

**Table 7:** *In silico* drug-likeness and the bioavailability of the RMEO compounds. (1) Sabinene, (2) limonene, (3) 2-nonanone, (4) 2-decanone, (5) 5-norbornene-2-methanol, (6) 2-undecanone, (7) 2-undecanol, (8) 2-dodecanone, (9) 2-undecanol acetate, and (10) caryophyllene.

Prediction	1	2	3	4	5	6	7	8	9	10
	Drug-likeness prediction									
Lipinski	Yes^∗^	Yes	Yes	Yes	Yes	Yes	Yes	Yes	Yes	Yes^∗^
Egan	Yes	Yes	Yes	Yes	Yes	Yes	Yes	Yes	Yes	Yes
Veber	Yes	Yes	Yes	Yes	Yes	Yes	Yes	Yes	Yes	Yes
Bioavailability score	0.55	0.55	0.55	0.55	0.55	0.55	0.55	0.55	0.55	0.55

^∗^1 violation: MLOGP > 4.15.

**Table 8:** *In silico* evaluation of the pharmacokinetic properties (ADME) of the RMEO compounds. (1) Sabinene, (2) limonene, (3) 2-nonanone, (4) 2-decanone, (5) 5-norbornene-2-methanol, (6) 2-undecanone, (7) 2-undecanol, (8) 2-dodecanone, (9) 2-undecanol acetate, and (10) caryophyllene.

Prediction	1	2	3	4	5	6	7	8	9	10
	ADME prediction									
*Physicochemical properties*										
TPSA (Å^2^)	0.00 Å^2^	0.00 Å^2^	17.07 Å^2^	17.07 Å^2^	20.23 Å^2^	17.07 Å^2^	20.23 Å^2^	17.07 Å^2^	26.30 Å^2^	0.00 Å^2^

*Absorption parameters*										
Water solubility (log mol/L)	−4.629	−3.568	−3.428	−4.068	−1.548	−4.684	−4.174	−5.267	−4.591	−5.555
Solubility class	Soluble	Soluble	Soluble	Soluble	Soluble	Soluble	Soluble	Soluble	Soluble	Soluble
Caco-2 permeability (log Papp in 10^−6^ cm/s)	1.404	1.401	1.488	1.487	1.48	1.486	1.472	1.486	1.609	1.423
Intestinal absorption (%)	95.35	95.89	94.69	94.34	90.02	94.00	91.48	93.66	93.59	94.84
Skin permeability log *K*_*p*_ (cm/s)	−1.342	−1.720	−1.350	−1.295	−2.379	−1.333	−1.397	−1.46	−1.81	−1.58

*Distribution parameters*										
VDss (log L/kg)	0.566	0.396	0.206	0.268	0.285	0.324	0.323	0.373	0.227	0.652
BBB permeability	Log BB > 0.30 Yes	Log BB > 0.30 Yes	Log BB > 0.30 Yes	Log BB > 0.30 Yes	Log BB > 0.30 Yes	Log BB > 0.30 Yes	Log BB > 0.30 Yes	Log BB > 0.30 Yes	Log BB > 0.30 Yes	Log BB > 0.30 Yes

*Metabolism parameters*										
CYP2D6 and CYP3A4 substrate	No	No	No	No	No	No	No	No	No	No
CYP2D6 and CYP3A4 inhibitors	No	No	No	No	No	No	No	No	No	No

*Excretion parameters*										
Total clearance log (mL/min/kg)	0.071	0.213	1.502	1.538	0.156	1.574	1.636	1.61	1.68	1.088
Renal OCT2 substrate	No	No	No	No	No	No	No	No	No	No

**Table 9:** Evaluation of the toxicological endpoints of the major compounds in RMEO. (1) Sabinene, (2) limonene, (3) 2-nonanone, (4) 2-decanone, (5) 5-norbornene-2-methanol, (6) 2-undecanone, (7) 2-undecanol, (8) 2-dodecanone, (9) 2-undecanol acetate, and (10) caryophyllene.

	Hepatotoxicity	Nephrotoxicity	Carcinogenicity	Immunotoxicity	Mutagenicity	Cytotoxicity	Predicted LD_50_ (mg/kg)	Class
1	Inact. (0.81)	Inact. (0.91)	Inact. (0.59)	Inact. (0.51)	Inact. (0.82)	Inact. (0.71)	5000	V
2	Inact. (0.76)	Inact. (0.88)	Inact. (0.65)	Inact. (0.95)	Inact. (0.97)	Inact. (0.82)	4400	V
3	Inact. (0.69)	Inact. (0.81)	Inact. (0.63)	Inact. (0.99)	Inact. (0.97)	Inact. (0.73)	5000	V
4	Inact. (0.69)	Inact. (0.81)	Inact. (0.63)	Inact. (0.99)	Inact. (0.97)	Inact. (0.73)	5000	V
5	Inact. (0.86)	Inact. (0.64)	Inact. (0.67)	Inact. (0.99)	Inact. (0.79)	Inact. (0.86)	4300	V
6	Inact. (0.69)	Inact. (0.81)	Inact. (0.63)	Inact. (0.99)	Inact. (0.97)	Inact. (0.73)	5000	V
7	Inact. (0.70)	Inact. (0.75)	Inact. (0.68)	Inact. (0.97)	Inact. (0.98)	Inact. (0.78)	1000	IV
8	Inact. (0.69)	Inact. (0.81)	Inact. (0.63)	Inact. (0.99)	Inact. (0.97)	Inact. (0.73)	5000	V
9	Inact. (0.57)	Inact. (0.59)	Inact. (0.51)	Inact. (0.86)	Inact. (0.99)	Inact. (0.65)	3000	V
10	Inact. (0.80)	Inact. (0.92)	Inact. (0.70)	Act. (0.54)	Inact. (0.95)	Inact. (0.75)	5300	V

^∗^Inact.: inactive, Act.: active, values between brackets are the probability of being active or inactive. Toxicity class explanation: Class IV: for substances that are harmful if swallowed (LD_50_ ranging from 300 to 2000 mg/kg); Class V: for compounds that may be harmful if swallowed (LD_50_ ranging between 2000 and 5000 mg/kg); Class VI: not harmful if swallowed (LD50 > 5000 mg/kg).

**Table 10:** The molecular free binding affinity (measured in kcal/mol) obtained from computational simulations of the identified compounds in RMEO.

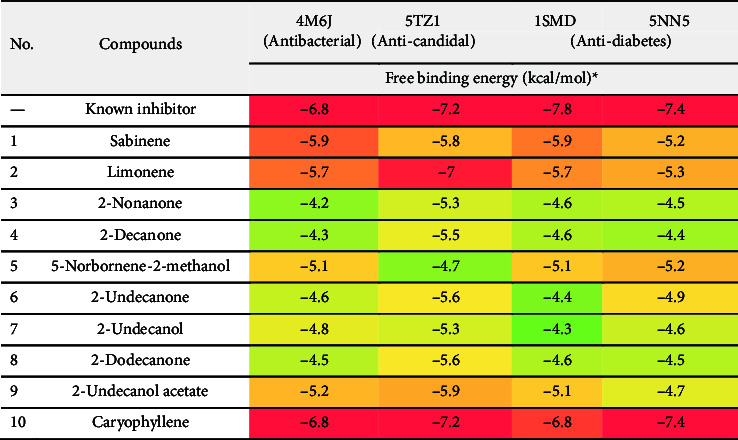

## Data Availability

Data are available on request from the authors.
